# Subacute lymphocytic thyroiditis after lobectomy in a patient with papillary thyroid carcinoma: a case report

**DOI:** 10.1186/1752-1947-7-3

**Published:** 2013-01-03

**Authors:** Young Sik Choi, You Jin Han, Go Eun Yeo, Su Kyoung Kwon, Bu Kyung Kim, Yo-Han Park, Sung Won Kim, Bong Kwon Chun, Eun Hee Kong, Jeong Hoon Kim

**Affiliations:** 1 Department of Internal Medicine, Kosin University College of Medicine, 262 Gamcheon Street SeoGu, Busan, 602-703, South Korea; 2 Department of Head and Neck Surgery, Kosin University College of Medicine, 262 Gamcheon Street SeoGu, Busan, 602-703, South Korea; 3 Department of Pathology, Kosin University College of Medicine, 262 Gamcheon Street SeoGu, Busan, 602-703, South Korea; 4 Department of Family Medicine, Kosin University College of Medicine, 262 Gamcheon Street SeoGu, Busan, 602-703, South Korea; 5 Department of General Surgery, Kosin University College of Medicine, 262 Gamcheon Street SeoGu, Busan, 602-703, South Korea

**Keywords:** Subacute lymphocytic thyroiditis, Papillary thyroid carcinoma, Thyroid lobectomy

## Abstract

**Introduction:**

Subacute lymphocytic thyroiditis is anautoimmune thyroid disease presenting with transient thyrotoxicosis as well as transient hypothyroidism. Several factors have been thought to be the initiating event in subacute lymphocytic thyroiditis. However, subacute lymphocytic thyroiditis that develops after thyroid lobectomy has not yet been reported in the literature. We report a case of subacute lymphocytic thyroiditis after lobectomy in a patient with papillary thyroid carcinoma.

**Case presentation:**

A 30-year-old Korean woman was referred to our center for thyroid tumor operation. She was diagnosed with suspicious papillary thyroid carcinoma by fine needle aspiration at a local medical clinic. The thyroid ultrasonography demonstrated a diffusely enlarged thyroid gland with a 0.4×0.3cm sized hypoechoic nodule in the left lobe. Left thyroid lobectomy by endoscopic thyroidectomy was performed via a transaxillary approach, and the nodule was confirmed to be a papillary thyroid carcinoma. On postoperative day 1, a thyroid function test revealed hyperthyroidism, and on postoperative day 8, a thyroid function test again revealed hyperthyroidism with decreased radioactive iodine uptake. Thyroid function tests showed euthyroid on postoperative day 48 and hypothyroidism on postoperative day 86. She was treated with levothyroxine.

**Conclusion:**

Subacute lymphocytic thyroiditis can develop after thyroid lobectomy. Thyroid autoantigen released during thyroid lobectomy may cause the onset or exacerbation of the destructive process.

## Introduction

Subacute lymphocytic thyroiditis is also known as silent sporadic thyroiditis and painless sporadic thyroiditis. It is clinically and pathologically similar to postpartum thyroiditis but occurs in the absence of pregnancy [1]. It accounts for one percent of all cases of hyperthyroidism [2]. It is considered a variant form of chronic autoimmune thyroiditis, suggesting that it is part of the spectrum of thyroid autoimmune disease [3].

Manipulation of the gland during thyroid biopsy or neck surgery, and especially during parathyroid surgery, can cause thyroiditis, manifested by transient neck pain and tenderness and transient hyperthyroidism [4-7]. However, subacute lymphocytic thyroiditis that develops after thyroid lobectomy has not yet been reported in the literature.

Recently, we had a case of subacute lymphocytic thyroiditis that developed after thyroid lobectomy in a patient with papillary thyroid carcinoma.

## Case presentation

**Figure 1:**
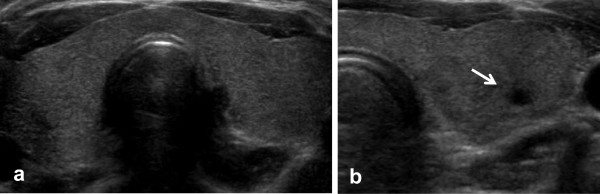
**(a) The transverse thyroid sonogram shows a diffusely enlarged thyroid gland with isoechoic and non-coarsened background parenchyma. (b)** A 0.4 × 0.3cm sized hypoechoic nodule (arrow) in the left thyroid.

**Figure 2:**
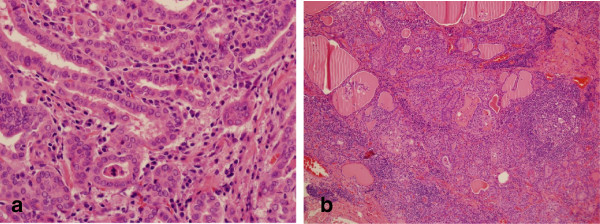
**Pathological finding reveals a papillary carcinoma. (a)** x400, hematoxylin and eosin stain and lymphocytic patches predominantly in peripheral portions of the thyroid parenchyma **(b)** ×100, hematoxylin and eosin stain.

A 30-year-old Korean woman was diagnosed with suspicious papillary thyroid carcinoma (PTC) by fine needle aspiration (FNA) at a local medical clinic. She was referred to our hospital for operation. She has no personal or family history of thyroid dysfunction and no specific medication history. Physical examination of neck revealed a diffusely enlarged and non-tender thyroid gland. There were no palpable cervical lymph nodes. US was carried out using a real time linear array 10-MHz transducer. The thyroid US demonstrated a diffusely enlarged thyroid gland with a 0.4×0.3cm sized hypoechoic nodule in the left thyroid (Figure 1). She wanted to diagnose the thyroid nodule again in our hospital. US-guided FNA was performed again on that nodule, and FNA cytology was diagnosed as suspicious PTC. We also examined for BRAF ^V600E^ mutation using FNA cytology materials. BRAF ^V600E^ mutation was identified by multiplex real time polymerase chain reaction assay using Anyplex™ BRAF V600E Real Time Detection (V2.0) (Seegene, Inc., Seoul, Korea), which allows for simultaneous amplification of total nucleic acid of V600E mutation of BRAF and internal control (human β globin gene). Computed tomography scan using contrast agent was done three weeks before operation. No abnormal finding was noted. The patient had no thyrotoxic symptoms during one month of the preoperative period. She underwent a left thyroid lobectomy by endoscopic thyroidectomy via a transaxillary approach for the thyroid tumor. The right thyroid was not manipulated during operation. Pathologic finding revealed a papillary carcinoma, and other portions of the thyroid parenchyma revealed lymphocytic patches predominantly in peripheral portions. There were rarely lymphoid follicles with germinal center (Figure 2).

**Figure 3:**
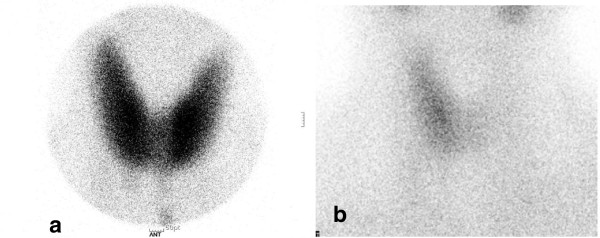
**(a) Preoperative Tc-99m scintigraphy checked at a local medical clinic shows normal uptake of the tracer. (b)** Postoperative Tc-99m scintigraphy shows faint visualization of the right thyroid lobe.

On laboratory examination, the preoperative laboratory test was performed one month before operation was within normal limits. The serial change of thyroid function tests and the titer of autoantibodies were summarized in Table 1. On postoperative day (POD) 1, she didn’t definitive complain. A thyroid function test (TFT) showed hyperthyroid with Antithyroglobulin antibody and thyroglobulin (Tg) (Table 1). On POD 8, the patient was consulted by the department of endocrinology. On physical examination, blood pressure was 120/80mmHg, and heart rate was 119beat/min and regular. Neck examination revealed no sign of tenderness and redness. TFT was still increased but Tg level was normalized (Table 1). Tc-99m scintigraphy showed faint visualization of the right thyroid lobes, and 24-hour radioactive uptake of ^131^I was 0.7% (Figure 3b), which suggested destructive thyroiditis. On POD 48, thyroid function tests revealed euthyroid. On POD 86, she complained of fatigue and weakness. A TFT revealed hypothyroidism (Table 1). The patient was treated with levothyroxine for her symptoms of hypothyroidism. On POD 140, follow-up TFT revealed mild hyperthyroidism (Table 1). The patient was treated with levothyroxine.

**Table 1:** The serial changes of thyroid function tests and titer of autoantibodies

	**Pre-OP**	**POD 1**	**POD 8**	**POD 48**	**POD 86**	**POD 140**	**Normal range**
T3 (ng/dL)	72.27	109.9	141.54	76.38	74.51	74.05	65-150
TSH (μIU/mL)	1.171	0.015	0.011	0.059	130.467	0.127	0.55-4.78
FT4 (ng/dL)	1.15	3.71	3.63	0.86	0.36	1.64	0.78-1.54
Tg (ng/mL)	54.48	390.0	63.78	ND	ND		1.4-78.0
Anti-Tg Ab (U/mL)	139.0	210.8	259.8	ND	69.9		< 60
Anti-MS Ab (U/mL)	172.1	ND	228.7	ND	707.3		< 60
TBII (U/L)	< 1	ND	< 1	ND	ND		< 10

Pre-OP, preoperative; POD, postoperative day; TSH, thyroid stimulating hormone; Tg, thyroglobulin; Anti-Tg Ab, antithyroglobulin antibody; Anti-MS Ab, antimicrosomal antibody; TBII, thyrotropin-binding inhibitory immunoglobulin; ND, not done.

## Discussion

Thyroiditis is an inflammation of the thyroid gland that has several etiologies. The various forms of thyroiditis can be divided into those associated with thyroid pain and tenderness, and those that are painless. Bindra et al. [1] classified painless thyroiditis according to etiology as Hashimoto’s thyroiditis, subacute lymphocytic thyroiditis, postpartum thyroiditis, drug-induced thyroiditis, and Riedel’s thyroiditis. The etiology of Hashimoto’s thyroiditis, subacute lymphocytic thyroiditis, postpartum thyroiditis, and lithium is autoimmune, and the etiology of drug-induced thyroiditis except lithium is inflammation [1].

Subacute lymphocytic thyroiditis affects four times more women than men, and the risk is increased in persons who live in areas of iodine sufficiency [3,8] About one half of patients with subacute lymphocytic thyroiditis present with a small goiter [9]. The present case is a Korean woman, and Korea is an iodine-sufficient area [10]. She also had a small goiter. In subacute lymphocytic thyroiditis, inflammation damages thyroid follicles and activates proteolysis of the Tg stored within the follicles. Therefore, the first biochemical change ininflammatory thyroiditis before the onset of thyrotoxicosisis an increase in the serum concentration of Tg [11]. Tg levels of present changes were also markedly increased on POD 1, and returned to the normal range on POD 8 (Table 1). This suggests that excreted Tg was normalized soon. In subacute lymphocytic thyroiditis, serum T4 concentrations are proportionally higher than T3 concentrations, reflecting the ratio of stored hormone in the thyroid gland [3]. Our case also showed normal-ranged T3 with increased FT4 levels. Serum antithyroid peroxidase concentrations are high in about 50% of patients at the time of diagnosis, but not to the extent that is found in Hashimoto’s thyroiditis [3,9]. The values may rise transiently in the following weeks and then decline, but they remain elevated after thyroid function returns to normal. In the present case, antithyroglobulin antibody and antimicrosomal antibody were both increased in the thyrotoxic period; however, antithyroglobulin antibody was normalized in the hypothyroid state, and antimicrosomal antibody was markedly increased in the hypothyroid state.

In subacute lymphocytic thyroiditis, the thyroid contains a lymphocytic infiltrate that partially resembles Hashimoto’s disease but without the fibrosis, Hürthle cells, and extensive lymphoid follicle formation [9,12]. Our case also showed lymphocytic infiltration on resected tissues without extensive lymphoid follicle formation. This suggested our case was chronic lymphocytic thyroiditis with papillary thyroid carcinoma and subacute lymphocytic thyroiditis that developed following thyroid lobectomy.

Two cases of thyroiditis with transient thyrotoxicosis were reported in patients who underwent parathyroid surgery for parathyroid adenoma [5]. In those cases, thyroid autoantibodies were not detected. Therefore, transient thyrotoxicosis could be secondary to intraoperative thyroid gland manipulation in those cases. However, our present case had goiter and thyroid autoantibodies before surgery. This suggested that thyroid autoantigen released during thyroid lobectomy may have caused the onset or exacerbation of the destructive process. One of the most common morbidities observed after thyroid lobectomy is hypothyroidism, with a reported incidence ranging from 10.9% to 42.6% [13-15]. However, subacute lymphocytic thyroiditis that develops after thyroid lobectomy has not been reported in the literature.

## Conclusions

To the best of our knowledge, this is first report of subacute lymphocytic thyroiditis that develops after thyroid lobectomy. Thyroid autoantigen released during thyroid lobectomy may cause the onset or exacerbation of the destructive process. If a patient with chronic autoimmune thyroiditis complains of mild thyrotoxic symptoms after thyroid lobectomy, thyroid function tests will be needed even in the early postoperative period.

## Consent

Written informed consent was obtained from the patient for publication of this case report and any accompanying images. A copy of the written consent is available for review by the Editor-in-Chief of this journal.

## Abbreviations

FNA: Fine-needle aspiration; US: Ultrasonography; PTC: Papillary thyroidcarcinoma; BRAF: B isoform of the Raf; POD: Postoperative day.

## Competing interests

The authors declare that they have no competing interests.

## Authors’ contributions

YC conceived the study and was principal writer of the manuscript. YH, GY, SK, YP, BC, EK, JK interpreted patient data, collected previously published literature on the subject, and contributed to writing the manuscript. SK performed the surgical procedure and helped to write the manuscript. All authors read and approved the final manuscript.
